# Many-body localization enables iterative quantum optimization

**DOI:** 10.1038/s41467-022-33179-y

**Published:** 2022-09-20

**Authors:** Hanteng Wang, Hsiu-Chung Yeh, Alex Kamenev

**Affiliations:** 1 School of Physics and Astronomy, University of Minnesota, Minneapolis, MN 55455 USA; 2 Shanghai Center for Complex Physics, School of Physics and Astronomy, Shanghai Jiao Tong University, 200240 Shanghai, China; 3 William I. Fine Theoretical Physics Institute, University of Minnesota, Minneapolis, MN 55455 USA

**Keywords:** Quantum information, Condensed-matter physics

## Abstract

Many discrete optimization problems are exponentially hard due to the underlying glassy landscape. This means that the optimization cost exhibits multiple local minima separated by an extensive number of switched discrete variables. Quantum computation was coined to overcome this predicament, but so far had only a limited progress. Here we suggest a quantum approximate optimization algorithm which is based on a repetitive cycling around the tricritical point of the many-body localization (MBL) transition. Each cycle includes quantum melting of the glassy state through a first order transition with a subsequent reentrance through the second order MBL transition. Keeping the reentrance path sufficiently close to the tricritical point separating the first and second order transitions, allows one to systematically improve optimization outcomes. The running time of this algorithm scales algebraically with the system size and the required precision. The corresponding exponents are related to critical indexes of the continuous MBL transition.

## Abstract (web summary)

There are several proposals for quantum algorithms solving optimisation problems, but so far none of them has provided a clear speedup. Here, the authors propose an iterative protocol featuring periodic cycling around the tricritical point of a many-body localization transition.

## Introduction

Optimization problems are ubiquitous^1,2^. A large subclass of them is discrete optimization tasks, which may be mapped onto spin models with the optimal solution being a ground state of a certain classical spin Hamiltonian. The optimization problems are hard due to the spin-glass phase^3–5^, i.e., the presence of multiple local minima in the energy landscape of the corresponding model. The idea of utilizing quantum tunneling in order to facilitate transitions between these local minima was coined a long time ago. Probably the earliest and most transparent way of doing it is realized via the adiabatic quantum annealing (QA) procedure^6–8^. Its bottleneck is associated with exponentially small energy gaps between instantaneous energy levels of the corresponding quantum Hamiltonian^9–15^. Those lead to Landau–Zener transitions^16–19^, which take the system out of its adiabatic ground state. As a result in order to succeed, the QA should be performed exponentially slow.

This stimulates interest in constructing approximate diabatic protocols^20,21^, collectively known as quantum approximate optimization algorithms^22–26^. The idea is to force the system to gradually approach its GS with relatively fast running cycles^27–30^. The goal of such algorithms is not finding the exact ground-state configuration, which corresponds to an NP-hard optimization problem but searching for a state within a given energy distance, *δ*_*ϵ*_, from the ground state.

**Fig. 1:**
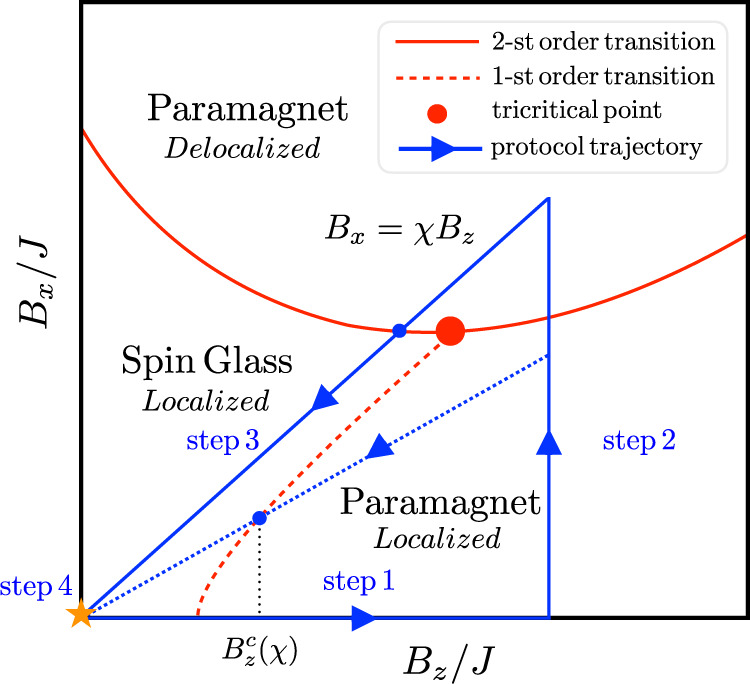
Phase diagram and the protocol. Phase diagram of the Hamiltonian (3) for a specific reference state. The full red line indicates a second-order transition^51^ between MBL and delocalized paramagnet; the dashed red line is the 1st order transition between the glass and MBL paramagnet. They meet at the tricritical point. The blue lines with arrows represent one optimization cycle. The dotted blue line is a subcritical slope, *χ* < *χ*_*c*_, which most likely brings the system back to the initial reference state.

Iterative version of optimization, which runs along a closed cycle in the space of parameters, turns to be efficient and has already appeared in the literature, see e.g., refs. 27–30. Combining it with the idea of the reference Hamiltonian^31–33^ leads to new protocol. The latter calls for using a control parameter (e.g., a longitudinal magnetic field) which is collinear with a local Bloch sphere direction of the individual qubits. The key observation is that, with the existence of reference Hamiltonian, the cycle must encircle a tricritical point^34,35^ of the many-body localization (MBL)^36–46^ transition. Here the MBL is understood as taking place in the many-body Hilbert space^36,43^, rather than in the real space^37,38^. The three phases coming together at the tricritical point are the spin glass, the MBL paramagnet, and the delocalized paramagnet, see Fig. 1.

In this work, we suggest an iterative quantum algorithm which runs along a closed cycle in the space of parameters. The cycle starts in the spin glass and goes successively into MBL and delocalized paramagnets before returning back to the spin glass, where the projective measurement is performed. This leads to an adjustment of the cycle parameters according to a result of the measurement taken at the end of the previous cycle. We show that such a strategy allows one to navigate the system arbitrarily close to the MBL tricritical point, as required by the proposed algorithm. As an example of optimization in a spin-glass system, we use Sherrington–Kirkpatrick (SK) model^47^ (a classical NP-hard problem^48^), whose MBL properties are discussed in refs. 41, 42, 44, 45.

The idea mimics a conventional refrigeration engine with the MBL transition in place of the exothermic condensation transition. We show that iterations of such cycle lead to a systematic decrease of energy of the measured state. Given a desired precision of the optimization, the cycle trajectory should pass increasingly close to the tricritical point. The cycle duration and the number of required cycles scale algebraically, both with the system size, *N*, and the desired precision, *δ*_*ϵ*_. The corresponding exponents are expressed through static and dynamic critical indexes of the MBL transition. Though we can not prove it, we conjecture that MBL critical exponents provide bounds on the performance efficiency of approximate optimization algorithms.

## Results

### Iterative quantum optimization protocol

As an example of an optimization problem with a glassy landscape, we choose a realization of the Sherrington–Kirkpatrick (SK) model^47^ specified by a Hamiltonian

1 $${H}_{{{{{{{{\rm{SK}}}}}}}}}=\mathop{\sum }\limits_{ij}^{N}{J}_{ij}{\sigma }_{i}^{z}{\sigma }_{j}^{z}.$$

Here ${\sigma }_{i}^{z}$ are *z*-Pauli matrices, which represent binary optimization variables, labeled by *i* = 1, 2, … *N*. The cost function is chosen to be quadratic in these parameters given by a cost matrix, *J*_*ij*_. In our examples its matrix elements are taken from independent Gaussian distributions with zero mean and variance *J*^2^/*N*. Eigenstates of the Hamiltonian, denoted as *α* = 1, 2, …, 2^*N*^, are encoded by bit-strings, $\{{s}_{i}^{\alpha }\}$, with ${s}_{i}^{\alpha }=\pm 1$ showing “up” or “down” polarization of the *i*th spin. The corresponding eigenenergies are ${E}_{\alpha }=\mathop{\sum }\nolimits_{ij}^{N}{J}_{ij}{s}_{i}^{\alpha }{s}_{j}^{\alpha }$. Since all the terms in the Hamiltonian (1) commute with each other, the problem is purely classical.

It is known^5,49,50^ that *E*_*α*_ form a glassy landscape with exponentially many local minima (i.e., states such that flipping any one (or even a few) spins results in energy being increased). Simulated classical annealing is typically trapped into one of such local minima. The local minima are separated from each other by the Hamming distance of the order *N* spin flips. The goal of the optimization is to find progressively deeper local minima, eventually hitting the global one.

The conventional adiabatic QA procedure calls for modifying the Hamiltonian (1) to add non-commutative (aka quantum) terms. The simplest of such quantum terms is (in general time-dependent) magnetic field applied in the *x*-direction:

2 $$H(t)={H}_{{{{{{{{\rm{SK}}}}}}}}}+{B}_{x}(t){H}_{{{{{{{{\rm{q}}}}}}}}},\qquad \,{H}_{{{{{{{{\rm{q}}}}}}}}}=-\mathop{\sum }\limits_{i=1}^{N}{\sigma }_{i}^{x}.$$

If the *x*-magnetic field is initiated to be large, *B*_*x*_ ≫ *J*, the ground state is close to all spins being polarized in the *x*-direction^42,44^. Such ground state is separated by a large gap, ∼ *B*_*x*_, from the rest of the spectrum. Cooling the system down to a temperature *T* ≪ *B*_*x*_ puts it almost surely in its true ground state. One then slowly decreases *B*_*x*_(*t*) down to zero so that the Hamiltonian goes back to the pure SK model (1). If this process is adiabatic, the state of the system follows its instanteneous ground state and arrives at the global SK minimum. For the system to not undergo any Landau–Zener transition, the annealing rate should be ${\tau }_{{{{{{{{\rm{anneal}}}}}}}}}^{-1}\; \ll \;{{{\Delta }}}_{\min }^{2}/{B}_{x}$, where ${{{\Delta }}}_{\min }$ is a minimal avoiding crossing gap, encountered by the ground state, and d*B*_*x*_(*t*)/d*t* ≈ *B*_*x*_/*τ*_anneal_. As argued in refs. 9–11 some of these gaps are exponentially small, demanding an exponentially long annealing time, *τ*_anneal_.

Hereby, we suggest an iterative cyclic algorithm capable of systematically approaching the ground state, while not being exponentially slow. Before the first cycle starts, one performs a simulated classical annealing, arriving at one of the local minima, which we will call a reference state, $\{{s}_{i}^{r}\}$. Each cycle consists of the four successive steps summarized in Fig. 1:

*Step 1*. The qubit array is initialized to the reference state and is programed to represent the following Hamiltonian

3 $$H(t)={H}_{{{{{{{{\rm{SK}}}}}}}}}+{B}_{x}(t){H}_{{{{{{{{\rm{q}}}}}}}}}+{B}_{z}(t){H}_{{{{{{{{\rm{ref}}}}}}}}}^{r},$$

where the *z*-field in the reference Hamiltonian is tailor-made to be co-directed with all the spins of the given reference bit-string, $\{{s}_{i}^{r}\}$,

4 $${H}_{{{{{{{{\rm{ref}}}}}}}}}^{r}=-\mathop{\sum}\limits_{i}{s}_{i}^{r}{\sigma }_{i}^{z}.$$

One starts from the pure SK model, *B*_*x*_ = *B*_*z*_ = 0, and then increases *B*_*z*_(*t*) from zero passing the critical field ${B}_{z}^{c}$, separating the spin-glass phase from the paramagnet. Since *B*_*x*_ = 0 in step 1, the Hamiltonian is purely classical and the system remains in the reference state, no matter how fast *B*_*z*_ is increased. In fact, all the states remain to be pure bit-strings of *H*_SK_, but their relative energies do change. The ${H}_{{{{{{{{\rm{ref}}}}}}}}}^{r}$ is chosen in a way to push the energy of the reference state sharply down: *E*_*r*_(*B*_*z*_) = *E*_*r*_(0) − *NB*_*z*_, since every spin in the reference state is aligned with the local *B*_*z*_ direction, by construction. The other local minima are far in the Hamming distance from the reference state and thus evolve typically as ${E}_{\alpha }({B}_{z})={E}_{\alpha }(0)\pm \sqrt{N}{B}_{z}$. As a result, soon enough the reference state is the unique ground state, separated by the gap. This first happens at the critical field ${B}_{z}^{c} \ \approx \ {\delta }_{\epsilon }$, where *δ*_*ϵ*_ = (*E*_*r*_(0) − *E*_GS_(0))/*N* is the currently achieved energy separation between the reference state and the exact ground state.

*Step 2*: *B*_*x*_ is increased while *B*_*z*_ is fixed. The gap in the paramagnetic phase is proportional to the total magnetic field $\sqrt{{B}_{x}^{2}+{B}_{z}^{2}}$, and is independent of the system size. One does not need an exponential or even a power law (in system size) long time to increase *B*_*x*_ while keeping the system in the ground state of the full Hamiltonian (3). However, since the full Hamiltonian is now quantum, its ground state is a superposition of many bit-string states. The *B*_*x*_ is increased until it reaches a certain ratio with the *z*-field: *χ* = *B*_*x*_/*B*_*z*_. Reaching large enough *B*_*z*_ in step 1 is crucial for the efficiency of the step 2. If one fails to cross the first order transition along step 1, the state of the system is located within a continuum of other states. It then undergoes uncontrollable Landau–Zener transitions within the spin-glass phase, resulting typically in a higher energy state.

*Step 3*: Decreasing *B*_*z*_ and *B*_*x*_ keeping the fixed ratio *χ* between them. Along this path, the system again crosses the phase boundary between the paramagnetic and the glassy phases. This boundary is marked by the first avoiding crossing transition between the ground state and the lowest excited state. The size of the corresponding gap strongly depends on the slope *χ*, which we discuss in detail in the next section. The upshot is that Landau–Zener transitions may occur during this part of the cycle, but with an overwhelming probability they leave the system in a state with an energy, which is lower than that of the initial reference state. The main danger is that the system remains in the reference state. This may be avoided, however, by a careful choice of the slope *χ*.

*Step 4*. After both *B*_*x*_ and *B*_*z*_ reach zero in the end of step 3, the system ends up in a superposition state. Now the measurement of each qubit is performed and the state collapses to a certain bit-string. Starting from this measured bit-string, the simulated annealing leads the system down to a nearest local minimum. If the energy of this new local minimum is less than that of the reference state, it is taken as the new reference state and the cycle is repeated from step 1. If, however, its energy is larger or the same, the system is initiated back to the old reference state and the cycle is again repeated from step 1.

Three key features of this protocol qualitatively improve its performance vis-a-vis the conventional QA. First, the reference state is iteratively set to be the minimal energy local minimum found in all previous trials. This way the reference energy never increases. Second, the choice of the reference Hamiltonian guarantees that Zener transitions in step 3 almost always decrease the energy. Third (and most significant), cycling around the tricritical point of the MBL transition allows to accomplish such energy decrease in a polynomial time. The second and the third items on this list are explained in the next section.

### MBL transition and the phase diagram

**Fig. 2:**
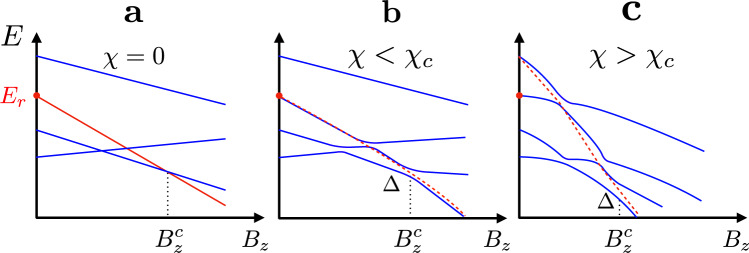
Spectrum and Landau–Zener transition. A sketch of the spectrum of Eq. (3) for different values of the slope *χ* = *B*_*x*_/*B*_*z*_. The red dots represent the SK energy of the reference state. **a** The red line depicts the energy of the reference state. **b**, **c** The reference state is the eigenstate only at *B*_*z*_ = *B*_*x*_ = 0. The red dashed line indicates a diabatic trajectory of step 3, undergoing Landau–Zener transition (from large *B*_*z*_ to small *B*_*z*_).

To illustrate the statements made above, consider Fig. 2 depicting schematically the energy spectrum of instanteneous Hamiltonian (3) vs. *B*_*z*_ for several fixed slopes *χ*, such that *B*_*x*_ = *χB*_*z*_. Figure 2a shows *χ* = 0 case, which corresponds to step 1 of the protocol. Since *B*_*x*_ = 0, the Hamiltonian is classical, and there are no transitions between the states. The corresponding energy levels cross each other. The reference Hamiltonian (4) is chosen in a way to ensure that the reference state (red line in Fig. 2a) goes down with a maximal slope. As a result, the reference state is destined to become a ground state at a certain critical field ${B}_{z}^{c}$. For ${B}_{z} \; > \; {B}_{z}^{c}$, there is a finite energy gap between the ground reference state and the rest of the spectrum. We thus refer to this phase as the paramagnet. Since all the states of such a paramagnet are represented by pure bit-strings, they are perfectly many-body localized in the bit-string basis. Notice that within the glassy phase, ${B}_{z} \; < \; {B}_{z}^{c}$, the reference state crosses only the states whose SK energy is less than *E*_*r*_.

Figure 2b shows the spectrum for *χ* < *χ*_*c*_. Due to the presence of *H*_q_, spin flips are allowed leading to avoiding crossings gaps in the spectrum. At small *B*_*x*_, these gaps are exponentially small, because of typically large (order *N*) Hamming distance between low-energy local minima. This makes the critical field ${B}_{z}^{c}(\chi )$ to be well defined in the large *N* limit. It marks the first order transition between MBL glass and MBL paramagnet phases. If the step 3 of the protocol is run (right to left) along this trajectory (dotted blue line in Fig. 1) with a non-exponentially small rate, the state of the system most likely follows the dashed red line. This brings the system back to the initial reference state, making the protocol fail. Notice, however, that in rare cases when the state does follow the adiabatic trajectories, the energy of the system is bound to be below the initial energy, *E*_*r*_.

To increase the probability of adiabatic transitions lowering the energy, the gaps need to be increased. This is achieved by working at *χ* > *χ*_*c*_, Fig. 2c. Such a strategy comes with a steep prize, however. Indeed, the reference state may also hybridize now with higher energy states. This leads to undesirable transitions increasing the energy (dashed red line in Fig. 2c). The question is if one can benefit from energy decreasing adiabatic trajectories, without being handicapped by Zener transitions to higher energy states (the latter phenomenon is responsible for the failure of the conventional QA^9–11,14,15^).

To answer this question, one needs to examine MBL transition on the phase diagram of our protocol, Fig. 1. Being defined by the Hamiltonian (3), the latter is tight to a specific reference state. Depending on the quantum component *B*_*x*_, this state and its neighbors may be either localized (small *B*_*x*_) or delocalized (large *B*_*x*_) in the bit-string basis. The transition between the two is characterized by a divergent localization–Hamming-length in the many-body Fock (i.e., bit-string) space^36–40^. Therefore the MBL transition is of the 2nd order^42,44,45,51^. It divides the phase space, Fig. 1, onto two disconnected regions. Notice that for any *B*_*x*_ there is the MBL transition at some energy within the many-body spectrum. The full red line in Fig. 1 refers to the MBL transition at (*B*_*z*_-dependent) energy of the reference state.

As explained above, there is also the 1st order transition between gapless^52^ (in the large *N* limit) spin-glass phase and the gaped paramagnet, both within the localized phase. The latter transition is not associated with a divergent Hamming distance. Since to the right of the 1st order transition line, the reference state is the ground state, this line terminates at a tricritical point somewhere along the 2nd order MBL transition boundary, Fig. 1.

Position of the tricritical point defines a critical slope *χ*_*c*_ of step 3 part of the cycle. For *χ* < *χ*_*c*_ step 3 encounters the 1st order transition within the MBL phase. Since all states below the reference one are many-body localized, the avoiding crossing gaps are exponentially small. Unless performed adiabatically (i.e., within exponentially long time), the step 3 is bound to bring the system back to its initial reference state.

The situation is qualitatively different for *χ* > *χ*_*c*_. Here the step 3 trajectory passes through the second-order transition from a delocalized paramagnet to a localized glass phase. Above the transition, the eigenstates are mixtures of resonances formed by bit-strings which are remote in hamming distance and spread around the Fock space. At the second-order phase transition point the avoiding crossing, defined by energy difference between the ground and the first excited state, exhibits algebraic finite-size scaling i.e., ${{\Delta }}\propto 1/{N}^{z/{d}_{{{{{{{{\rm{eff}}}}}}}}}}$^14^. This is a direct consequence of the divergent localization–Hamming length at the MBL transition^53^. We use *z*/*d*_eff_ notation for the corresponding critical exponent, having in mind that Δ ∝ *ξ*^−*z*^, while for a finite-size system the localization length at the transition is $\xi \sim {N}^{1/{d}_{{{{{{{{\rm{eff}}}}}}}}}}$, where *d*_eff_ is an effective dimensionality of the many-body Hilbert space. Once entering into the spin-glass phase, the states become localized, and subsequent avoiding crossings scale exponentially ${{\Delta }}\propto \exp \{-Nf({B}_{x})\}$, where *f*(*B*_*x*_) goes to zero coming to the transition from below. Thus away from the finite-size vicinity of the transition, the tunneling events are suppressed. The essence of the algorithm is utilizing the adiabatic transitions within this small region near the localization transition.

The avoiding crossing gap also depends on the slope *χ*. The second-order gap closing is terminated at the tricritical point *χ*_*c*_, below which the transition is of the first order and the gap scales exponentially. When approaching *χ*_*c*_ from above, we define the critical exponent *θ* describing the gap closing while approaching *χ*_*c*_ as,

5 $${{\Delta }}\propto \frac{{(\chi -{\chi }_{c})}^{\theta }}{{N}^{z/{d}_{{{{{{{{\rm{eff}}}}}}}}}}},$$

According to numerical estimate of ref. 54, *z*/*d*_eff_ ≈ 0.6 for SK model. For Hopfield model (a cousin of SK), *z*/*d*_eff_ = 1/3^14^.

Therefore if step 3 is performed within the power-law time, ${\tau }_{3} \sim {{{\Delta }}}^{-2} \sim {N}^{2z/{d}_{{{{{{{{\rm{eff}}}}}}}}}}$, it results in a certain number of the avoiding crossing transitions taking the adiabatic turn. What remains to be shown is that these transitions indeed lead to a systematic energy decrease, not overshadowed by transitions to the higher energy states, as in Fig. 2c. The key insight is that this may be achieved by tuning the slope *χ* closer to the critical one from above, $\chi \to {\chi }_{c}^{+}$.

**Fig. 3:**
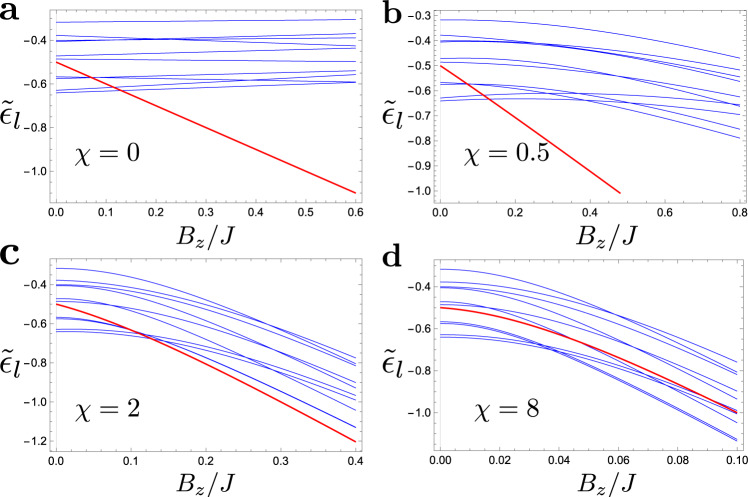
Spectra of isolated local minima. Spectra of isolated local minima, shown in blue, for **a** *χ* = 0, **b** *χ* = 0.5, **c** *χ* = 2, and **d** *χ* = 8, within the same realization. As *χ* increases, a progressively larger fraction of higher energy states curves down (due to the level repulsion from their local Hamming distance neighborhood) to intersect the reference state.

**Fig. 4:**
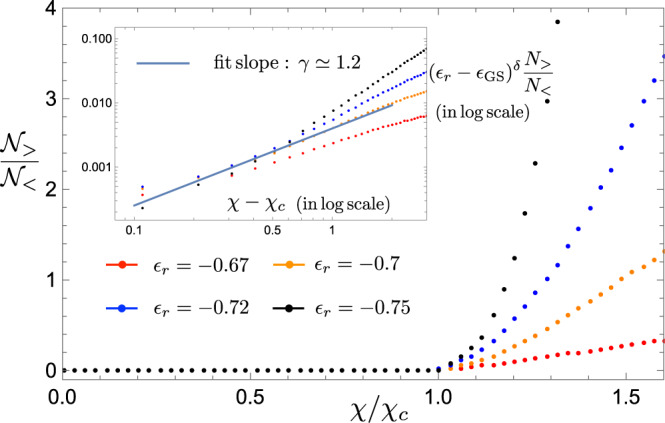
States ratio and scaling exponents. Ratio of higher- and lower-energy isolated local minima intersecting with the reference state as a function of the slope *χ*/*χ*_*c*_. The data is taken for several values of the reference state energy *ϵ*_*r*_. The ground-state energy is *ϵ*_GS_ = −0.8. The inset shows ${({\epsilon }_{r}-{\epsilon }_{{{{{{{{\rm{GS}}}}}}}}})}^{\delta }{{{{{{{{\mathcal{N}}}}}}}}}_{ { > }}/{{{{{{{{\mathcal{N}}}}}}}}}_{{ < }\,}$ vs. *χ* − *χ*_*c*_ in log-log scale; solid line is fitting with Eq. (6).

To show this we numerically isolate local minima states along with their simulated annealing basins of attraction from other local minima basins. One may diagonalize Hamiltonian (3) in each of such basins (details of this procedure are described in “Methods”). This way we keep the geometry of the levels, undisturbed by avoiding crossings generated by tunneling between the local minima. It allows us to track exact identities of all local minima, in particular the reference state. Figure 3 shows energies of such isolated local minima vs. *B*_*z*_. One can now calculate the number of local minima, with both higher energy, ${{{{{{{{\mathcal{N}}}}}}}}}_{{ > }}$, and lower energy, ${{{{{{{{\mathcal{N}}}}}}}}}_{{ < }}$, crossing the reference state. Figure 4 shows the ratio ${{{{{{{{\mathcal{N}}}}}}}}}_{ { > }}/{{{{{{{{\mathcal{N}}}}}}}}}_{{ < }}$ vs. slope *χ*. As expected, for *χ* < *χ*_*c*_ there are practically no higher energy states getting in contact with the reference one. On the other hand, the fraction of the higher energy states grows rapidly for *χ* > *χ*_*c*_. The smaller the energy of the reference state the faster this fraction grows. This is expected since, for a deep local minimum, there are not too many other local minima below it, but there are plenty above. The most important lesson from Fig. 4 is what the ratio grows continuously as

6 $$\frac{{{{{{{{{\mathcal{N}}}}}}}}}_{ { > }\,}}{{{{{{{{{\mathcal{N}}}}}}}}}_{{ < }\,}}\propto \frac{{(\chi -{\chi }_{c})}^{\gamma }}{{({\epsilon }_{r}-{\epsilon }_{{{{{{{{\rm{GS}}}}}}}}})}^{\delta }},$$

where *γ* ≈ 1.2 and *δ* ≈ 2.0 are critical exponents (see inset in Fig. 4) and *ϵ*_*α*_ = *E*_*α*_/(*NJ*). The critical slope can depend on the reference state. In our simulations, this dependence appears to be very weak, if any, with *χ*_*c*_ ≈ 3.6.

Equations (5) and (6) allow to estimate efficiency of the algorithm vis-a-vis its running time, precision, and other requirements. First one fixes the desired precision, i.e., the energy deviation from the global minimum: *δ*_*ϵ*_ = (*E*_*r*_(0) − *E*_GS_(0))/*N* = *ϵ*_*r*_ − *ϵ*_GS_. For simplicity, let us settle with the regime where every other cycle, in average, results in lowering the energy of the reference state. This amounts to an equal number of upper and lower-energy local minima intersections, ${{{{{{{{\mathcal{N}}}}}}}}}_{ { > }}/{{{{{{{{\mathcal{N}}}}}}}}}_{{ < }\,}=1$. This dictates that the protocol should be run at $\chi -{\chi }_{c} \; \lesssim \;{\delta }_{\epsilon }^{\,\delta /\gamma }$. Although it requires a more and more precise knowledge of *χ*_*c*_, if precision is increased, the good news is that the required *χ* − *χ*_*c*_ does not scale with the system size. We discuss ways of “on the fly” measurement of *χ*_*c*_ in next section.

The running time is given by a number of required cycles, *n*_*c*_, multiplied by duration of the step 3, *τ*_3_ (steps 1, 2 and 4 are typically faster). The latter is given ${\tau }_{3}\; \gtrsim \; {{{\Delta }}}^{-2}\propto {N}^{2z/{d}_{{{{{{{{\rm{eff}}}}}}}}}}{\delta }_{\epsilon }^{-2\theta \delta /\gamma }$. Finally, assuming that every successful cycle eliminates a fraction *p* < 1 of remaining lower-energy states (see Supplementary Note 1 for details), one may estimate a number of required cycles as ${n}_{c} \sim N/|\log (1-p)|$. This leads to the total optimization time, which scales as

7 $$\tau={n}_{c}{\tau }_{3}\propto {N}^{2z/{d}_{{{{{{{{\rm{eff}}}}}}}}}+1}\,{\delta }_{\epsilon }^{\,-2\theta \delta /\gamma }.$$

This is our central result. It shows the algebraic scaling of the approximate optimization time with the system size and the desired precision. Importantly the exponents are expressed through those of the MBL transition. It is possible that MBL critical indexes provide hard bounds, which no approximate algorithm can exceed.

A recent study^55^ proposed a classical approximate message-passing algorithm with the duration *N*^2^*C*(*δ*_*ϵ*_) (with an unspecified function *C*(*δ*_*ϵ*_)). Our algorithm can match the performance of ref. 55, if 2*z*/*d*_eff_ ≤ 1. It can’t exceed it vis-a-vis *N*-scaling, since each cycle includes simulated annealing with the required time *τ*_sa_ ~ *N*. This limits the total duration by *N* ⋅ *τ*_sa_ ~ *N*^2^. Notice, however, that such *N*^2^ part is independent on *δ*_*ϵ*_ and therefore is not a bottleneck for *δ*_*ϵ*_ → 0. On the other hand, Eq. (7)," provides the *N*-scaling in this limit.

## Discussion

We have outlined the quantum approximate optimization algorithm, which is capable of systematically approaching the global minimum of glass within the power-law (in the system size) time (7). It is based on a variant of the quantum annealing, with the reference state-specific Hamiltonian (3) and the iterative cycle encircling the tricritical point of the MBL transition. Though SK model is used here for illustration purposes, we expect the algorithm to be applicable to a wider class of discrete optimization tasks with the continuous transition from the gapless spin-glass phase to the polarized (gapped) paramagnet (as function of *B*_*x*_). While it is known to be the second order in many models (e.g., SK, Hopfield^14^), there are instances where this is the first order transition. Examples are provided by the *p*-spin models with large (possibly infinit) *p* (approaching random-energy universality class)^42,44^. In such cases, the algorithm will not succeed. Moreover, the proposed algorithm essentially relies on a single 2nd order phase transition separating the localized and delocalized regimes, as opposed to a sequence of transitions with intermediate “ergodic non-extensive” phases in between. Recent works on random regular graphs^56^ and SYK_4_ + SYK_2_ model^57^ indeed support the single transition scenario. There is no guarantee, however, that all discrete optimization problems belongs to this class and therefore our algorithm may fail in cases which do not.

An attractive feature of the algorithm is that it does not require an exceedingly long qubit coherence time. Indeed, the projective measurement is done after every cycle. Therefore the required coherence time scales as a period of the single cycle, ${\tau }_{3}\propto {N}^{2z/{d}_{{{{{{{{\rm{eff}}}}}}}}}}$. Moreover, if one or a few qubits produce a faulty readout, it will be automatically corrected by simulated classical annealing, performed after every quantum state measurement. Another advantage is a limited number of the required dynamical control parameters. In fact, after the Hamiltonian (3) is set, all qubits are subject to only two dynamically varying controls: *B*_*x*_(*t*) and *B*_*z*_(*t*). There is also the measurement step, requiring a simultaneous measurement of all ${\sigma }_{i}^{z}$.

The algorithm does not provide an exact solution of NP-hard problem. Such solution would require reaching exponentially small *δ*_*ϵ*_ and thus an exponentially long time. The main bottleneck is step 3 annealing, which requires algebraically long (in *δ*_*ϵ*_) time. On the other hand, iterative determination of *χ*_*c*_ does not constitute a significant overhead on the algorithm performance. To get approximate location of *χ*_*c*_, one can bound it from above and below. The lower bound is obtained from returning to the initial reference state. The upper bound is determined from repeated measurements of higher energy states distant from the reference one. By tuning *χ* half way between the two bounds and repeating the process, the number of trials scales logarithmically with the desired precision $\propto|\log ({\delta }_{\epsilon })|\sim \log N$.

## Methods

### Local minima isolation

Here, we discuss a phenomenological approach to numerically isolate local minima states along with their simulated annealing basins of attraction from other local minima basins. The low-energy Landau–Zener transitions occur only between the local minima states, $\left|l\right\rangle$, due to the fact that local minima are repelled down by their Hamming distance neighbors. To simplify the spectrum in the spin-glass phase, one may identify a basin state $\widetilde{\left|l\right\rangle }$, which is a wave packet localized at local minimum state $\left|l\right\rangle$, i.e., it is a superposition of $\left|l\right\rangle$ and its Hamming-neighbor states. Upon simulated annealing, all these states lead to the corresponding local minimum state, i.e., $\widetilde{\left|l\right\rangle }\to \left|l\right\rangle$. Therefore in the spin-glass phase, one can approximate the spectrum of Eq. (3) by the spectrum of local minima.

A hopping between any two local minima is typically exponentially small, since the Hamming distance is of the order of the system size *N*. An effective Hamiltonian between two basin states is

8 $${H}_{l,{l}^{\prime}}^{{{{{{{{\rm{block}}}}}}}}}=\left(\begin{array}{cc}{\tilde{E}}_{l}&{t}_{l{l}^{\prime}}\\ {t}_{l{l}^{\prime}}&{\tilde{E}}_{{l}^{\prime}}\end{array}\right),$$

where ${t}_{l{l}^{\prime}}$ is the effective hopping between *l* and ${l}^{\prime}$ basin states with energy ${\tilde{E}}_{l}$ and ${\tilde{E}}_{{l}^{\prime}}$, which is renormalized by the Zeeman effect of *B*_*z*_ and by repulsion from local Hamming neighborhood due to *B*_*x*_, i.e.,

9 $${\tilde{E}}_{l}={E}_{l}+{{{\Sigma }}}_{l}({B}_{z},{B}_{x}).$$

Here, Σ_*l*_(*B*_*z*_, *B*_*x*_) is the self-energy which gives the energy curves ${\tilde{\epsilon }}_{l}={\tilde{E}}_{l}/(NJ)$ of Fig. 3 without the anti-crossing effect.

**Fig. 5:**
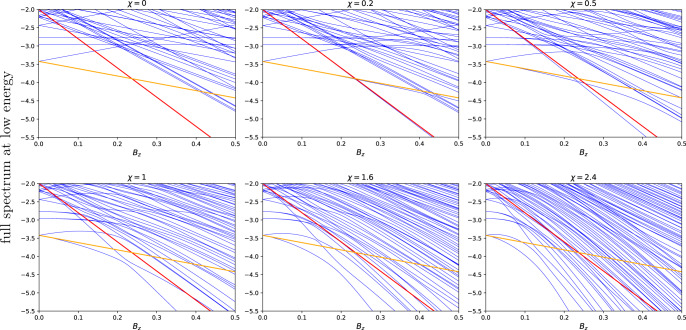
Exact diagonalization. Low-energy spectra of Eq. (3) for different *χ*. System size is *N* = 10. Red and orange straight lines are the reference and ground-state energies, correspondingly at *χ* = 0. They are repeated on all panels as a guide for eye. When *χ* = 0, energies are straight lines with slopes (11). For larger *χ*, energies are curved down, due to the level repulsion. Simultaneously the gaps open up.

By analyzing a small system-size exact diagonalization, shown in Fig. 5, we found that the self-energy is well approximated by

10 $${{{\Sigma }}}_{l}({B}_{z},{B}_{x})=NJ\left[{f}_{l}-\sqrt{{({f}_{l}+{m}_{l}{B}_{z}/J)}^{2}+{({B}_{x}/J)}^{2}}\right],$$

where *m*_*l*_ and *f*_*l*_ are basin-dependent phenomenological parameters discussed below. This expression interpolates between the limiting cases of *χ* ≪ 1 and *χ* ≫ 1. For *χ* ≪ 1 one may put *B*_*x*_ = 0, finding Σ_*l*_ = − *m*_*l*_*NB*_*z*_. The corresponding slope, *m*_*l*_, for a given local minimum *l* measures the spin configuration overlap with the reference state *r*:

11 $${m}_{l}=\frac{1}{N}\mathop{\sum }\limits_{i=1}^{N}{s}_{i}^{l}\cdot {s}_{i}^{r}=1-2{d}_{l}/N,$$

where *d*_*l*_ is the Hamming distance from the reference state to the basin *l*. We found that *d*_*l*_’s are distributed according to a binomial distribution

12 $$P({d}_{l})=\frac{1}{{2}^{N}}\left(\begin{array}{l}N\\ {d}_{l}\end{array}\right),$$

which is natural, if one assumes totally random spin flipping (or not) to reach another local minimum.

For *χ* ≫ 1, one may start assuming *B*_*z*_ = 0. At *B*_*x*_/*J* ≪ 1, Eq. (10) is approximate by

13 $${{{\Sigma }}}_{l}{\approx} - {N}{B}_{x}^{2}/(2{f}_{l}J).$$

This may be viewed as a result of the second order, in *B*_*x*_, perturbation of the SK model. The energy of the local minimum goes down due to the level of repulsion, and the second-order perturbation comes from the one-spin flip states. The factor 1/(2*f*_*l*_*J*) describes the average inverse energy difference between the local minimum and one-spin flip states. The distribution of *f*_*l*_’s is approximated by a uniform box in the interval 1/4 < *f*_*l*_ < 3/4. Finally at *B*_*x*_ ≫ *J*, the system is fully polarized with Σ_*l*_ ≈ − *NB*_*x*_. Equation (10) is the simplest way to interpolate between all these limits, which works extremely well for small system-size simulations.

To perform larger system-size simulations, leading to Fig. 4, we statistically generate multiple local minima energy curves according to Eqs. (9)–(13). The distribution of SK local energies, *E*_*l*_ is taken from refs. 49, 50 and is assumed to be statistically independent from the other random parameters, *m*_*l*_ and *f*_*l*_. We simulated system sizes up to *N* = 200 and verified that the qualitative features of Fig. 4 are robust against variations in specific distributions of the random parameters. The first order (red dashed) line in Fig. 1 is determined by the position of $({B}_{z}^{c}(\chi ),\; \chi {B}_{z}^{c}(\chi ))$ for a fixed reference state, while ${B}_{z}^{c}(\chi )$ is given by the last intersection of the reference state.

## Supplementary information

Supplementary Information

## Data Availability

The data used to create the plots are available from the authors upon reasonable request.

## Code availability

The numerical codes to obtain the data are available from the authors upon reasonable request.
