# Publisher Correction: Many-body localization enables iterative quantum optimization

**DOI:** 10.1038/s41467-022-33655-5

**Published:** 2022-10-06

**Authors:** Hanteng Wang, Hsiu-Chung Yeh, Alex Kamenev

**Affiliations:** 1 School of Physics and Astronomy, University of Minnesota, Minneapolis, MN 55455 USA; 2 Shanghai Center for Complex Physics, School of Physics and Astronomy, Shanghai Jiao Tong University, 200240 Shanghai, China; 3 William I. Fine Theoretical Physics Institute, University of Minnesota, Minneapolis, MN 55455 USA

**Keywords:** Quantum information, Condensed-matter physics

**Correction to**: *Nature Communications* 10.1038/s41467-022-33179-y, published online 20 September 2022

The original version of this Article contained an error in Equation 8 in the PDF version. The right hand side was written as a single matrix element instead of a two-by-two matrix, and incorrectly read:

$${H}_{l,{l}^{\prime} }^{block}=({\tilde{E}}_{l}{t}_{l{l}^{\prime}}\,{t}_{l{l}^{\prime}}\,{\tilde{E}}_{{l}^{\prime}})$$

The correct form of Equation 8 is:

$${H}_{l,{l}^{\prime}}^{{{{{{{{\rm{block}}}}}}}}}=\left(\begin{array}{cc}{\tilde{E}}_{l}&{t}_{l{l}^{\prime}}\\ {t}_{l{l}^{\prime}}&{\tilde{E}}_{{l}^{\prime}}\end{array}\right)$$

This has been corrected in the PDF version of the Article. The html version was correct and has not been changed.

