# Supplementary material for: Many-body localization enables iterative quantum optimization
Source: Nat Commun. 2022 Sep 20;13:5503. doi: 10.1038/s41467-022-33179-y (PMC9489738; doi:10.1038/s41467-022-33179-y)
Supplement: Supplementary file 1 — Supplementary Information [file 41467_2022_33179_MOESM1_ESM.pdf]

Supplementary Information for
"Many-body localization enables iterative quantum optimization"

Hanteng Wang, Hsiu-Chung Yeh, and Alex Kamenev

Supplementary Note 1: Decreasing energy and elimination of states

In this section, we demonstrate how energy decreased in each iteration. As discussed in the main text, we propose an iterative protocol to reduce the energy of the SK model under four consecutive steps. At step 3, the reference state meets an anti-crossing which defines the phase boundary at a particular χ. We can investigate what state/energy the reference state will be scattered on if the Landau-Zener transition is forbidden at the phase boundary.

We utilize the phenomenological model in "Methods" section in the manuscript. The scaled energies of local minima depend on B_z and $B_x = \chi B_z$ as follows:

$$\tilde{\epsilon}_l = \epsilon_l + J\left[f_l - \sqrt{(f_l + m_l B_z/J)^2 + (B_x/J)^2}\right]. \tag{1}$$

For a given χ with a particular realization of SK random couplings, the phase boundary is defined at the largest B_z that the local minimum l crosses reference state. By adiabaticity at phase boundary in step 3 of protocol, the system is scattered to the local minimum l with energy ϵ_l at the end of cycle since the subsequent scattering in glass phase is strongly suppressed. Hence, we simulate the distribution of energy ϵ_r^{new} after one cycle in Fig. 1 with different initial reference energy ϵ_r. If one starts with a reference state (e.g. $\epsilon_r = -0.65$ in dashed red), the system will reach some energy $\epsilon_r^{\text{new}} = \epsilon$ with probability distribution $Pr(\epsilon)$ (solid red curve). The probability distributions are approximately bounded by the reference energy since we run simulation in the regime $\chi \gtrsim \chi_c$. This constraint indeed comes from the construction of reference state and Hamiltonian.

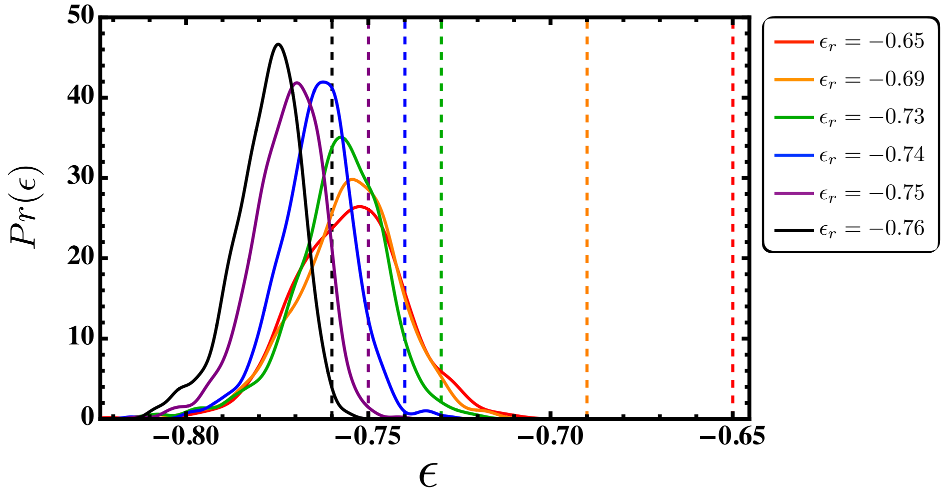

Supplementary Figure 1. **Probability distribution of scattered energy.** The system is scattered from colored dashed energy to the distribution with same color. In the simulation, we use $N = 150$ and $\chi \approx \chi_c$ with 700 realizations. The ground state energy is around -0.8.

The most important feature in Fig. 1 is that the distributions shift to lower energy as the reference energy goes down. This encourages to decrease the reference energy cycle by cycle. In thermodynamic limit, the energy value, ϵ_r^{new}, peaking at $Pr(\epsilon)$ is the typical energy, which is non-exponentially close to the initial ϵ_r, see Fig. 2. As a consequence, any finite precision $\delta\epsilon$ are reachable within algebraically large iterations.

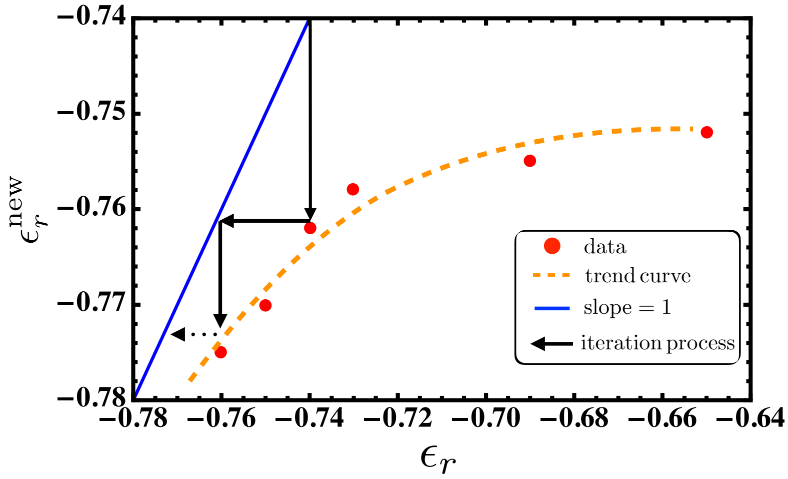

Supplementary Figure 2. **Iteration process and energy decreasing.** For each ϵ_r, the typical scattered energy ϵ_r^{new} is the peak of $Pr(\epsilon)$ in Fig. 1. These forms the red dots data with the orange dashed qualitative trend curve. The blue line is $\epsilon_r^{\text{new}} = \epsilon_r$, which corresponds the physical meaning of setting scattered state as a new reference state. The black arrows depict how reference energy changes in cycles: energy decreases after annealing (downward arrow) and new energy is the input in next cycle (leftward arrow).
